# Reinfections with Different SARS-CoV-2 Omicron Subvariants, France

**DOI:** 10.3201/eid2811.221109

**Published:** 2022-11

**Authors:** Nhu Ngoc Nguyen, Linda Houhamdi, Léa Delorme, Philippe Colson, Philippe Gautret

**Affiliations:** Aix Marseille University, Marseille, France; Institut Hospitalo-Universitaire Méditerranée Infection, Marseille

**Keywords:** COVID-19, respiratory infections, severe acute respiratory syndrome coronavirus 2, SARS-CoV-2, SARS, coronavirus disease, zoonoses, viruses, coronavirus, reinfection, Omicron, subvariants, France

## Abstract

We describe 188 patients in France who were successively infected with different SARS-CoV-2 Omicron subvariants, including BA.1, BA.2, and BA.5. Time between 2 infections was <90 days for 50 (26.6%) patients and <60 days for 28 (14.9%) patients. This finding suggests that definitions for SARS-CoV-2 reinfection require revision.

In Belgium, 96 cases of early SARS-CoV-2 reinfection were reported during December 1, 2021–March 10, 2022; the cases had a median of 47 days (range 17–65 days) between 2 positive samples (*1*). Five of those cases indicated primary infections with Omicron subvariant BA.1, followed by Omicron BA.2 reinfections. In addition, we previously reported that the reinfection risk with Omicron was 6-fold higher than with other SARS-CoV-2 variants (*2*). In this study, we describe cases of COVID-19 reinfection with different Omicron subvariants after a primary infection with Omicron subvariants BA.1 or BA.2 in Marseilles, France. We performed real-time reverse transcription PCR and next-generation genomic sequencing of nasopharyngeal swab samples to identify subvariants as previously described (*3*). We retrospectively retrieved patient age and gender information from electronic medical files and anonymized data before analysis. We identified reinfected patients by using a computerized alert system that focused on samples with primary Omicron BA.1 or BA.2 subvariant infections followed by reinfection with any Omicron subvariant. This study was approved by the ethics committee of the University Hospital Institute Méditerranée Infection (approval no. 2022–029). Access to patient biologic and registry data in the hospital information system was approved by the data protection committee of Assistance Publique-Hôpitaux de Marseille and recorded in the European General Data Protection Regulation registry (no. RGPD/APHM 2019–73).

We identified 188 (0.7%) cases of reinfection out of 27,972 patient samples that tested positive for the SARS-CoV-2 Omicron variant during November 28, 2021–July 22, 2022. Of those 188 patients, 181 were first infected with the Omicron BA.1 subvariant and were reinfected as follows: 82 patients with Omicron BA.2, 14 patients with Omicron BA.4, 84 patients with Omicron BA.5, and 1 patient with a BA.1 and BA.2 recombinant subvariant (XAC recombinant lineage) (Appendix Figure). Three of the 181 patients infected with Omicron BA.1 were reinfected 2 times; for the first reinfection, they were infected with Omicron BA.2 and, for the second reinfection, they were infected with Omicron BA.5. In addition, 7 patients were first infected with the Omicron BA.2 subvariant, after which 1 patient was reinfected with the Omicron BA.4 subvariant, and 6 patients were reinfected with the Omicron BA.5 subvariant. 

Patients were 1–83 (median 32) years of age at the time of their second infection, and 131 (69.7%) were women. The median time between the primary and secondary infections was 146 days (range 7–214 days); median time between infection with Omicron BA.1 and reinfection with BA.2 was 84 days and for a primary infection with Omicron BA.1 and reinfection with BA.5 was 171 days (Appendix Figure). Among the 188 patients infected first with Omicron BA.1 or BA.2, the time between the primary and secondary infections was 1–29 days in 6 (3.2%) cases, 30–44 days in 4 (2.1%) cases, 45–59 days in 18 (9.6%) cases, 60–74 days in 10 (5.3%) cases, 75–89 days in 11 (5.8%) cases, and >90 days in 139 (73.9%) cases. In 50/188 (26.6%) patients, time between the 2 infections was <90 days and, in 28/188 (14.9%) patients, the time was <60 days between the 2 infections.

Our findings indicate that the time between confirmed primary infections and reinfections with different Omicron subvariants is frequently shorter than the 90-day definition of reinfections used by the US Centers for Disease Control and Prevention (*4*). Furthermore, the time can be shorter than the 60-day definition of reinfections used by the European Centre for Disease Prevention and Control (*5*). Similar to our findings, a study from Denmark reported 47 Omicron BA.2 reinfections that occurred 20–60 days after a primary BA.1 infection (M. Stegger et al., unpub. data, https://www.medrxiv.org/content/10.1101/2022.02.19.22271112v1). 

The first limitation of our study is that the number of cases was small. Second, we cannot exclude that some cases might have been concurrent infections with different subvariants, notably in the 3 cases that had a 7-day interval between the detection of 2 subvariants. In Marseille, the short time between emergence of different Omicron subvariants might have favored co-infections with different subvariants circulating within the population (Figure). Co-infections can be missed if the quantitative PCR has inadequate sensitivity, and whole-genome sequencing might fail to detect a variant with low prevalence in a patient. Finally, because most reinfection cases were identified from samples transferred to our laboratory by external entities, we were unable to describe COVID-19 vaccination and clinical status of the patients. Nonetheless, our results suggest that the currently used definitions for SARS-CoV-2 reinfection require revision with regard to the duration between primary and secondary infections.

**Figure:**
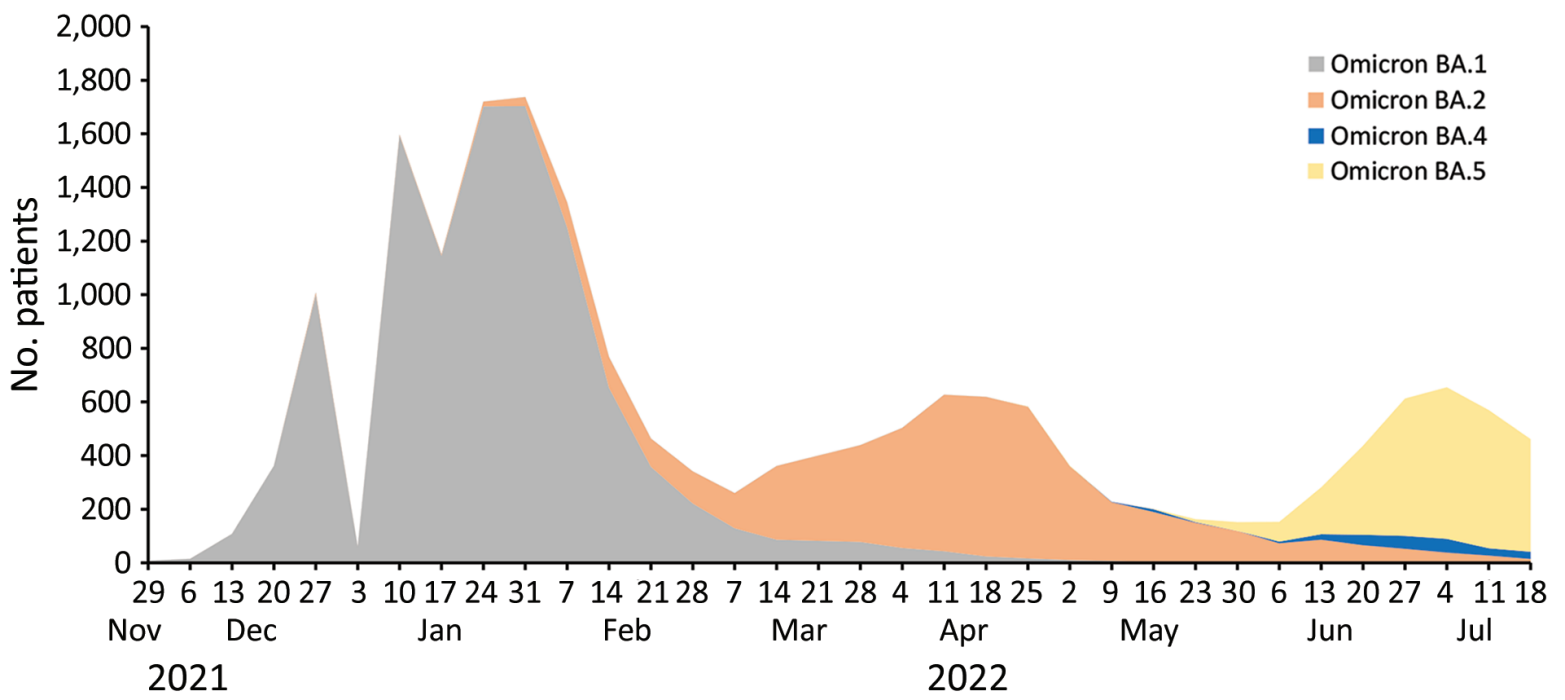
Number of patients with infections from different SARS-CoV-2 Omicron subvariants, France, November 28, 2021–July 22, 2022. Overall dynamics of infections with Omicron subvariants BA.1, BA.2, BA.4, and BA.5 are shown for cases diagnosed at the Institut Méditerannée Infection, Marseille, France. We performed real-time reverse transcription PCR and next-generation genomic sequencing of nasopharyngeal swab samples to identify Omicron subvariants BA.1, BA.2, BA.4, and BA.5. A total of 27,972 patient samples tested positive for Omicron subvariants.

Appendix Additional information for reinfections with different SARS-CoV-2 Omicron subvariants, France.
