# Supplementary material for: Reinfections with Different SARS-CoV-2 Omicron Subvariants, France
Source: Emerg Infect Dis. 2022 Nov;28(11):2341–3. doi: 10.3201/eid2811.221109 (PMC9622229; doi:10.3201/eid2811.221109)
Supplement: Appendix — Additional information for reinfections with different SARS-CoV-2 Omicron subvariants, France. [file 22-1109-Techapp-s1.pdf]

Article DOI: https://doi.org/10.3201/eid2811.221109

# Reinfections with Different SARS-CoV-2 Omicron Subvariants, France

## Appendix

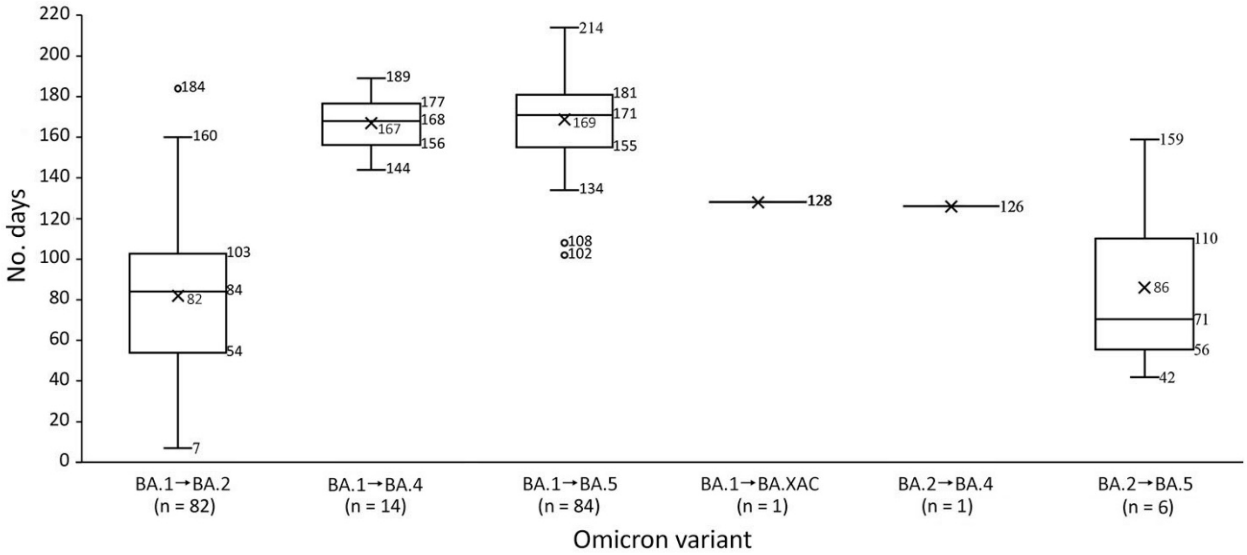


**Appendix Figure.** Number of days between primary infection and reinfections with different SARS-CoV-2 Omicron subvariants, France. Box plots indicate the number of days between 2 episodes of Omicron SARS-CoV-2 infections in 188 patients from Marseilles, France. The x axis indicates the primary infection with either Omicron subvariant BA.1 or BA.2 followed by reinfection with a different Omicron subvariant. The vertical line inside the box represents the median, and the mean is marked with an X. The ends of the boxes are the lower and upper quartiles that represent observations outside the 25–75 percentile range. The error bars show the minimum and maximum values. Data falling outside the upper and lower quartile range are plotted as outliers.
